# Effect of parent-focused interventions for screen use on developmental outcomes in young children: a systematic review and meta-analysis

**DOI:** 10.1186/s12966-026-01919-8

**Published:** 2026-05-20

**Authors:** Amanda Machell, Carrie Ewin, Sharon Horwood, Katherine L Downing, Kylie D. Hesketh

**Affiliations:** 1 Institute for Physical Activity and Nutrition (IPAN), Deakin University, Geelong, VIC Australia; 2 School of Psychology, Deakin University, Geelong, VIC Australia

**Keywords:** Screen context, Early childhood, Cognitive development, Social-emotional development, Physical development.

## Abstract

**Introduction:**

Parents are a key target for interventions promoting healthy screen use in young children, but no review has evaluated the effect of such parent-focused interventions on developmental outcomes in early childhood. The objective of this study was to conduct a systematic review and meta-analyses of the effects of parent-focused interventions for screen use on young children’s social-emotional, cognitive, and physical development, as well as proximal indicators of development including sleep and physical activity. A secondary objective was to assess their impact on children’s screen use.

**Methods:**

Eight databases were searched with publication dates between 2007 and March 2025. Randomised controlled trials of parent-focused interventions targeting screen use in children (birth-5.99 years) were included if they reported outcomes related to both child screen use and development. Stata was used to undertake meta-analyses using multilevel random effects models to estimate effects for outcomes with data from at least three papers.

**Results:**

In total, 10 studies (11 papers) were eligible for inclusion, with eight studies (*n* = 1,776 participants) providing sufficient data to be included in meta-analyses of at least one outcome. Interventions targeted screen use duration (*n* = 8 studies), screen use before bed (*n* = 1 study) and screen content (*n* = 1 study; 2 papers). Interventions achieved reductions in social-emotional problems (ES=-0.32 (95%CI:-0.51,-0.12)), externalizing behaviors (ES=-0.31 (95%CI:-0.56,-0.05)) and screen time (ES=-0.92 (95%CI:-1.66,-0.18)). Few interventions (*n* = 2) targeted aspects of screen use other than duration (e.g. content quality and timing). Larger effects were achieved for interventions that were underpinned by a theory and incorporated a range of established behavior change techniques, compared to interventions that did not incorporate these aspects. Few studies explored intervention effects on physical activity, body mass index, cognitive development and/or motor skills, and heterogenous sleep outcomes were reported, making it difficult to draw definitive conclusions regarding these outcomes.

**Conclusions:**

Few parent-focused interventions for screen use were identified and most targeted screen use duration. Future intervention studies may consider targeting aspects of screen use other than duration (e.g. content quality), and evaluating their feasibility and acceptability for parents, compared to interventions targeting screen use duration. The protocol was prospectively registered on PROSPERO (ID: CRD420250654905).

**Supplementary Information:**

The online version contains supplementary material available at 10.1186/s12966-026-01919-8.

## Background

Young children (0–5 years of age) are growing up in an increasingly digital world [1, 2], with parents reporting excessive screen time as their top health concern for their children [3]. Despite these concerns, a recent (2025) US survey reported that 62% of children owned a tablet and 10% owned a smart phone by age six years [4]. The widespread availability of screen-based devices has raised concerns about their possible impacts on development - especially during the critical early childhood years (under age 6), a period marked by rapid brain growth and heightened neuroplasticity [5]. While screen use research had typically focused on the negative consequences of screen use [6, 7], there may also be both beneficial impacts on early development [8]. However, the specific aspects of screen use that contribute to beneficial developmental outcomes remains poorly understood.

Excess screen use has been linked with a range of negative developmental outcomes, including cognitive (e.g. language delays) [6], social-emotional (e.g. externalising problems) [9] and physical development (e.g. body mass index, motor skills) [7], as well as proximal indicators of healthy development, including sleep [10] and physical activity [11]. However, emerging evidence indicates some screen use, depending on context (e.g. timing, content, co-use), may have benefits for early childhood development [8]. Screen time guidelines from the World Health Organisation [12] and individual nations (e.g. Australia [13] and Canada [14]) recommend children aged under two years avoid screen use, and children aged two to five years engage in no more than one hour of screen time per day. Despite these recommendations, globally, the majority of children engage in more screen time than recommended: approximately 25% of children aged under two years, and 36% of children aged two to five years meet the respective screen time guidelines [15]. Paediatric societies of the US, UK and Canada, have moved beyond setting time limits, instead recommending families choose high quality programming, avoid violent content, and avoid screen use in the hour before bedtime [16–18].

Parents play an important role in influencing how, when, and why young children use screens, and are therefore, considered a key intervention target to improve the screen use behaviors of their children. A conceptual model by Morowska and colleagues [19] posits modifiable parental factors, including parent modelling (e.g. parents’ own screen use), parenting practices (e.g. allowing screen use before bed) and parent self-efficacy (e.g. to manage their child’s screen use), influence children’s screen use. Therefore, parent-focused interventions that target modifiable parental factors offer a promising approach to simultaneously improve children’s screen use and developmental outcomes, as well as their proximal indicators including improved sleep (e.g. through removing screens before bedtime) [20] and increased physical activity (e.g. through displacement) [21].

Prior reviews have explored associations between modifiable parental factors and screen use [22], and the effect of parent-focused interventions on screen use [10, 23] and sleep [10] in early childhood. Collectively, findings highlight the potential positive influence of modifiable parenting practices and related factors on children’s screen use. Those reviews indicated: (a) greater parental and mealtime screen use were associated with higher child screen use, whereas higher levels of parental self-efficacy, and the implementation of household screen use rules were associated with lower screen use among children aged less than six years (*n* = 87 studies) [22]; (b) educating parents on the importance of limiting screen time, providing alternative activities to screen use, and removing screens from children’s bedrooms were effective strategies for decreasing screen time among children aged two to five years (*n* = 6 studies) [23]; and (c) interventions delivered to parents and/or children (*n* = 5) were effective in reducing screen time (− 0.25 (95%CI: −0.42, − 0.08); *p* = 0.004)) but not effective for increasing sleep duration (0.09 (95%CI: −0.04, 0.22); *p* = 0.15) among children aged two to six years [10]. However, those reviews were limited by their lack of recency, with searches conducted more than five years ago [10, 23], and their limited scope, focusing on intervention effects on screen time duration [22, 23] and/or sleep [10], without considering other developmental outcomes or aspects of screen other than duration.

To our knowledge, no systematic review has explored the effect of parent-focused interventions for screen use on a range of developmental outcomes in early childhood. The objective of this systematic review was to expand on the current evidence by examining the effect of parent-focused interventions for screen use on a broad range of developmental outcomes, including their proximal indicators (i.e. sleep and physical activity) in early childhood (age 0-5.99 years). A secondary objective was to determine the effect of parent-focused interventions on screen use behaviors. Feasibility outcomes were also explored.

## Methods

### Study design and registration

This systematic review is reported in accordance with the Preferred Reporting Items for Systematic Reviews and Meta-Analyses (PRISMA) statement [24] and the JBI Manual for Evidence Synthesis for Systematic Reviews of Effectiveness. The PRISMA checklist is provided in Additional file 1.

### Eligibility criteria

The population, intervention, comparison, outcomes and study type (PICOS) framework was used to develop the inclusion criteria as follows: *Population*: typically developing children with a mean age less than six years at baseline. Studies involving children with a mean age six years or older at baseline or children with medical conditions (diagnosed with a condition which may affect growth, development or behavior such as Autism Spectrum Disorder or Attention Deficit Hyperactivity Disorder) were excluded. *Intervention*: parent-focused interventions targeting screen use in the home setting, either on its own or as part of a multi-component intervention, were considered (providing intervention effects due to changes in screen use could be isolated). Interventions targeting screen use in the education setting (e.g. childcare, preschool) were excluded because most screen use occurs in the home in this age group [19] and parents do not have control over screen use in education settings. *Comparator*: a group of children not exposed to a parent-focused intervention for screen use. *Outcome*: effects on both screen use of the child and at least one developmental outcome (or proximal indicator) must be reported. *Study design*: Only studies employing a randomised controlled trial (RCT) study design were included, as RCTs are considered the gold standard when assessing causal relationships. Quasi experimental studies, observational studies, conference abstracts, dissertations, grey literature and study protocols were excluded.

### Information sources

MEDLINE, PsycINFO, ERIC and CINAHL (via EBSCO), Scopus, Cochrane library, Informit Health Collection, and Google Scholar were searched in March 2025. We also screened reference lists of included studies to identify additional eligible studies.

### Search strategy

Search strategies were developed with guidance from an academic librarian, and employed keywords, subject headings and MeSH terms related to screen use, parenting, intervention, and early childhood. Search terms related to screen use and early childhood were adapted from a previously published systematic review of associations between screen use and early childhood development [8]. Outcome terms related to child development were not searched as searching outcomes has been shown to impact retrieval of relevant studies [25]. Database searches were limited to articles published in English and from 2007, corresponding to the debut of iPhones and the beginning of widespread use of smartphones [26]. The complete search strategy is available in Additional File 2.

### Selection process

Identified studies were imported to Covidence systematic review software (Veritas Health Innovation, Melbourne, Australia; available at www.covidence.org) and duplicates were removed. Following this, Covidence’s RCT Classifier tool was used to identify and exclude non-RCTs (99.5% sensitivity) [27]. Titles and abstracts were independently screened in duplicate by six reviewers (AM, KH, SH, KD, KP, BU) to identify articles for full text screening. Full text screening of the eligible articles was conducted in duplicate (AM, CE) to determine eligibility for inclusion in the review. Conflicts over study inclusion were resolved by discussion with a third reviewer (KH) until consensus was reached. Almost perfect raw agreement was achieved during title and abstract screening (κ = 0.27; range = 0.02 to 0.63; 98% raw agreement), however, the overall Kappa value was low. This appears due to three pairs of reviewers where the total number of papers screened was relatively low (*n* = 44–84), and each pair had only 1–2 papers indicated for potential inclusion but disagreed on all (total of 5 paper disagreements) which interferes with how Kappa is calculated. Almost perfect agreement was achieved during full text screening (κ = 0.85; 97% raw agreement).

### Risk of bias assessment

Risk of bias was assessed using the Joanna Briggs Institute (JBI) Revised Critical Appraisal Checklist for randomized controlled trials [28]. This tool provides a structured framework to evaluate potential sources of bias across key domains, including randomization procedures, allocation concealment, blinding, and the comparability of groups at baseline. Each item on the checklist was rated as “Yes,” “No,” or “Unclear”. One reviewer (AM) conducted the assessments, with another reviewer (CE) checking each criteria for each study for accuracy, and discrepancies resolved through discussion with a third reviewer (KH).

### Data extraction

One reviewer (AM) extracted data using a structured data extraction form in Excel, tailored to prompt retrieval of relevant information, with a second reviewer (CE) checking for accuracy. Details extracted included general study characteristics (e.g. year of publication, country where the study was conducted, study design and sample size), participant characteristics (age, sex), intervention details (theory and behavior change techniques, intervention focus and content, duration, dose, delivery mode, delivery setting, provider, assessment timepoints and comparison group), outcome measures and study findings.

### Statistical analysis

Meta-analysis was conducted for outcomes assessed in at least three studies. Standardized mean differences (Cohen’s *d*) were extracted directly from studies [29, 30] or calculated based on post intervention means and standard deviations using the “Practical Meta Analysis Effect Size Calculator” (Campbell Collaboration). Where multiple post-intervention time-points were reported, data from the time-point closest to intervention completion were used to ensure consistency in effect size calculations. The corresponding authors of five studies were contacted via email with one follow-up attempt made, requesting the post intervention means and standard deviations rather than: (a) screen time stratified by TV and smartphone viewing duration (0–30 min; 31–59 min; ≥60 min) [31]; (b) screen time reported as median and interquartile range [32]; (c) mean screen time duration only (i.e. standard deviation was not reported) [29]; (d) regression co-efficient for emotional problems score and physical activity [33]; or (e) extrapolating from figures where data were reported inconsistently across timepoints for zBMI and screen use (6, 12, 18 and 24 months) [34]; one response was received [32]. As the results for the remaining four studies could not be accurately converted to the required format [29, 31, 33] or accurately extrapolated from the provided figures (34), and to maintain methodological consistency, these studies were unable to be included in the corresponding meta-analyses but were retained in the narrative review.

Meta-analyses were performed using Stata, with random effects specified due to expected heterogeneity between studies. For studies reporting more than one outcome within an outcome domain, multi-level meta-analysis using three-level random effects models with random intercepts for study and for outcomes nested within studies were used to account for nesting of multiple effect sizes within individual studies. Heterogeneity was assessed with the *I*^*2*^ statistic using the following cut-offs: 25% = “small heterogeneity”; 50% = “medium heterogeneity”; and 75% = “large heterogeneity” [35]. Standardised classifications for the magnitude of effect were used: 0.20 representing a small effect, 0.50 representing a medium effect, and 0.80 representing a large effect [36]. Sub-group analyses and publication bias were unable to be assessed formally due to the small number of included studies (< 10 studies included in the meta-analyses) [37]. Forrest plots were used to display results for each meta-analysis.

### Deviations from the registered protocol

Several minor deviations from the original study protocol were made to enhance the comprehensiveness and feasibility of the review. First, the MEDLINE database was included in the initial search phase, as it had been unintentionally omitted during protocol development. Second, while the protocol originally specified duplicate data extraction, in practice, one author (AM) performed the initial data extraction, and a second author (CE) independently verified the accuracy and completeness of the extracted data. This approach for data extraction and risk of bias assessment is explicitly endorsed in several systematic review handbooks [38] and has been used in similar studies [39]. Third, although the protocol focused on studies reporting developmental outcomes, during the screening process we broadened the inclusion criteria to incorporate proximal indicators, specifically sleep and physical activity, as these are known to be closely related to cognitive, social-emotional and physical development in early childhood [40–42], therefore providing a more comprehensive exploration of intervention effects Fourth, the original protocol specified inclusion of studies involving children aged less than six years at baseline. During screening, this criterion was refined to include studies where the mean age of participants at baseline was less than six years, allowing the inclusion of studies with a broader age range. In one study [34], mean ages were reported separately for the intervention group (mean age = 5.8 years) and control group (mean age = 6.1 years) but not for the total sample. Given the similar group sizes (intervention group = 36; control group = 34), the average of the two group means (5.95 years) was calculated, and used to determine eligibility, and the study was retained. Finally, data analyses were conducted in Stata, rather than RevMan as planned, due to the extracted data requiring multi-level meta-analysis which is not supported in RevMan.

## Results

### Study selection

**Fig. 1:**
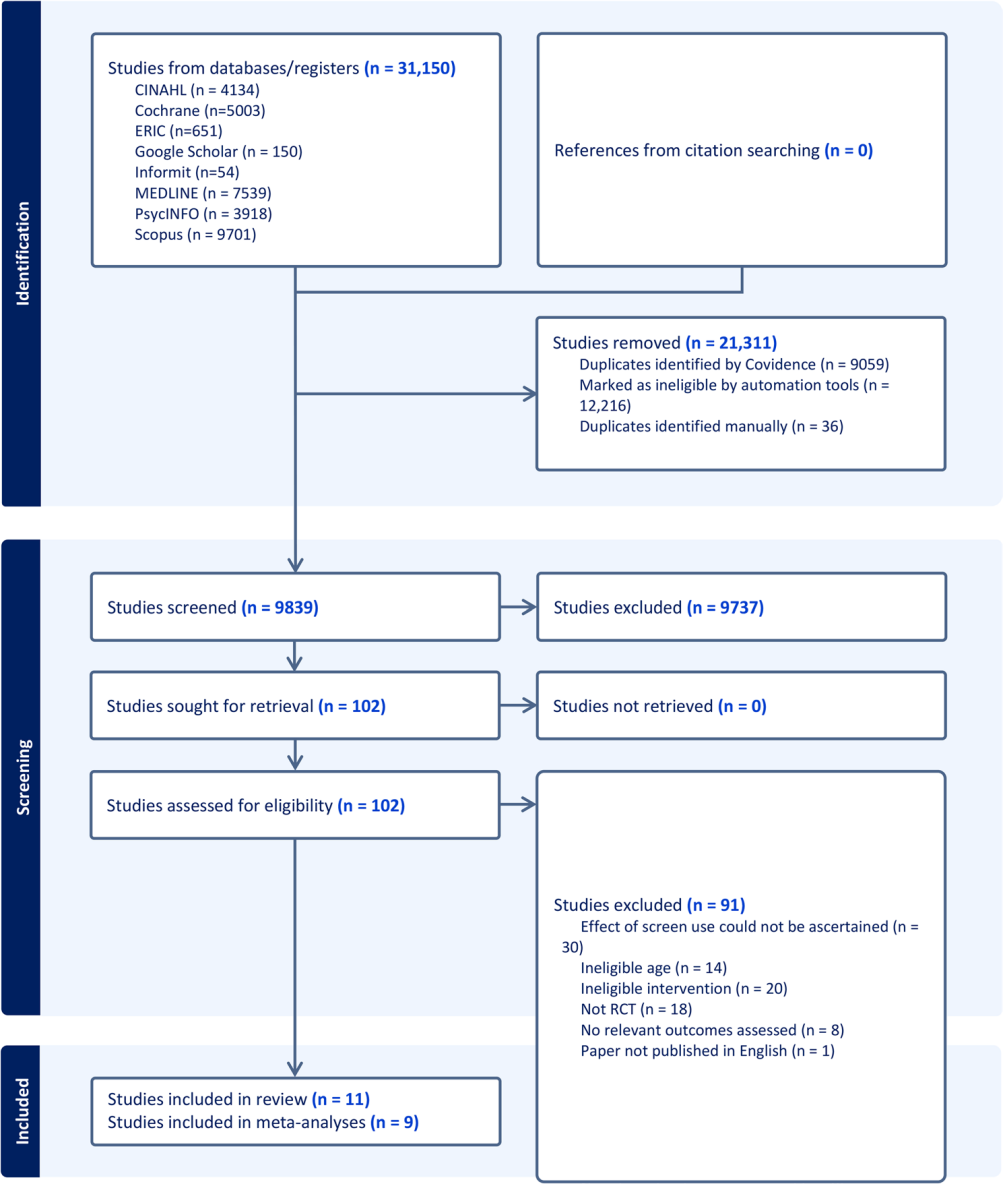
PRISMA flow diagram

A total of 31,150 records were retrieved. After removal of duplicates (*n* = 9,095) and removal of non-RCTs using Covidence’s RCT classifier tool (*n* = 12,216), 9,839 titles and abstracts were reviewed against the eligibility criteria, from which 102 full-text articles were retrieved and reviewed for inclusion. Reasons for exclusion at the full-text stage were: the effect of screen use could not be ascertained (*n* = 30); ineligible age, i.e., > 6 years at baseline (*n* = 14); ineligible intervention, e.g. did not target parenting (*n* = 20); not RCT (*n* = 18); no relevant outcomes assessed (*n* = 8); or the paper was not published in English (*n* = 1). No additional papers were identified from searching reference lists of the included studies. Finally, 10 studies (11 papers) were included in this review, with eight studies (*n* = 1,776 participants) providing sufficient data to be included in meta-analyses of at least one outcome (Fig. 1). Table 1 provides an overview of the results.

**Table 1:** Overall results summary

Outcome domain	Studies included (*n*)	Studies in meta-analysis (*n*)	Effect sizes (k)	Effect direction	ES	95% CI	I^2^
Social-emotional							
* All domains combined*	6	5	13	+	-0.32	-0.51,-0.12	62%
* Externalizing behaviors*	5	4	7	+	-0.31	-0.56,-0.05	75%
* Internalizing behaviors*	2			o			
* Social competence*	2			o			
* Global social-emotional development*	2			+			
**Cognitive**							
* Attention*	1			-			
* Problem solving*	1			+			
* Communication skills*	1			-			
**Physical**							
* Body mass index*	3			o			
* Motor skills*	1			o			
**Proximal indicators**							
* Physical activity*	3			o			
* Sleep*	5			o			
**Screen time**	10	6	6	+	-0.92	-1.66, -0.18	97%

+ positive effect

- no effect

o mixed effect

### Study characteristics

Characteristics of the included studies are reported in Table 2. Studies were conducted in the US [29, 34, 43], India [32, 33] Turkey [31, 44], Australia [45], Canada [46], UK [30] and Taiwan [47]. Participant age was reported as either a mean or a range. Mean ages spanned from 17 months [31] to 5.95 years [34], while studies reporting age ranges included children between nine months [32] and five years [33]. Sample sizes ranged from 22 [45] to 565 participants [29, 43]. Intervention effects were reported across a broad range of developmental outcomes including social-emotional [29, 30, 32, 33, 44, 47], physical (measured zBMI) [34, 44, 46], and cognitive development [30, 32], as well as proximal indictors of development including sleep [30, 31, 33, 43, 47] and physical activity [33, 34, 45]. Across all studies, social-emotional [29, 30, 32, 33, 44, 47] and motor skill [32] outcomes were parent-reported. For other outcomes, a mix of parent-report and objective measures were used. In one study (out of two), cognitive outcomes were objectively assessed [30]; in three (out of three) studies, zBMI was calculated from measured height and weight [34, 44, 46]; in two (out of three) studies physical activity was objectively assessed [34, 45]; and in one (out of five) studies sleep was objectively assessed [30].

**Table 2:** Characteristics of included studies

Study (country)	Mean age (Range)	*N*	Summary of intervention effect (measure used)									
			Cognitive	Social-emotional development				Physical dev.		Proximal indicators		Screen use
				Global Soc-Emo.	Social comp.	Int. prob.	Ext. prob.	Growth	Motor skills	PA	Sleep	
Birken, 2012* (46) (Canada)	IV: 3.12 yrs; C: 3.08 yrs	132						No effect (measured BMI)				No effect on weekday, weekend screen time (PQ)
Bulduk, 2025 (31) (Turkey)	17 mo.	166									↑ total, nighttime, daytime, & total daily sleep duration; ↓ awakenings, sleep latency, and number of naps (BISQ & CSHQ)	↓ TV, smartphone (PQ)
Christakis* 2013 (29) (US)	IV: 50.9 mo. C: 51.6 mo. (3–5 years)	565		↑ social competence & behavior overall score at 6 & 12 mo. (SCBE)	↑ social competence subscale score at 6 & 12 mo. (SCBE)	No effect on internalising behavior subscale score (SCBE)	↓ externalising behavior subscale score at 6 & 12 mo. (SCBE)					↑ prosocial & ↓ violent content at 6 mo; no effect at 12 mo. (PQ)
Garrison 2012 (43) (US)											↓ sleep problems at 6, 12, 18 mo. (CSHQ)	
Epstein, 2008* (34) (US)	IV: 5.8 yrs. C: 6.1 yrs. (4–7 years) Total sample: 5.95 years ^a^	70						↓ 6 & 12 mo. (measured BMI) & no effect at 18 & 24 mo.		No effect on counts/min (Actigraph)		↓ TV & computer at 6, 12, 18, 24 mo. (TV allowance device)
Hinkley, 2015* (45) (Australia)	IV: 2.94 yrs C: 2.85 yrs	22								NS % time stepping (ActivPAL)		No effect on total EM use (Time use diary)
Kaur, 2024* (33) (India)	2–5 yrs	340					No effect on emotional problems at 2 & 6 mo. (CBCL)			↑ PA at 2 & 6 mo. (PrePAQ)	No effect on sleep problems at 2 & 6 mo. (SDSC)	↓ screen time duration at 2 mo; no effect at 6 months (DESQ)
Lin, 2021* (47) (Taiwan)	5.6 yrs	129				↓ Internalising problems. (PSCL)	↓ externalising problems; ↓ att. prob. (PSCL)				↑ sleep quality (CSHQ)	↓ screen time (PQ)
Pickard, 2024* (30) (UK)	23.7 mo.	105	No effect on attention (Visual Search Task)				No effect on effortful or inhibitory Control (ECBQ)				No effect on sleep duration, awakenings & efficiency (Actigraphy Watch) No effect on sleep onset latency (BISQ)	↓ screen time in the hour before bed (PQ)
Poonia, 2024* (32) (India)	9–10 mo.	120	No effect on communication; ↑ problem solving (ASQ-3)	↑ social-emotional development (ASQ: SE2)	No effect on personal/ social (ASQ-3)				No effect on gross motor; ↑ fine motor skills (ASQ-3)			↓ screen time (PQ)
Yilmaz, 2015* (44) (Turkey)	IV: 3.52 yrs; C: 3.49 yrs (2–6 yrs)	363					↓ aggressive & delinquent behavior at 9 mo. (CBCL)	No effect (measured BMI)				↓ media time at 2, 6, 9 mo. (PQ)

*included in meta-analysis

^a^ calculated the average of the two (intervention and control) group means

*Abbreviations*: *PQ *Parent Questionnaire (tool not explicitly named), *BISQ *Brief Infant Sleep Questionnaire, *CSHQ *Children's Sleep Habits Questionnaire, *SCBE *Social Competence and Behavior Evaluation, *SDSC *Sleep Disturbance Scale for Children, *PSCL *Paediatric Symptom Checklist, *ECBQ *Early Childhood Behavior Questionnaire, *ASQ3 *Ages and Stages Questionnaire, *ASQ:SE2 *Ages and Stages Questionnaire, Social Emotional, *DESQ *Digital Screen Exposure Questionnaire, *CBCL *Child Behavior Checklist, *BMI *Body Mass Index, *EM *Electronic Media, *PA *Physical activity, ⭣: significant decrease, ⭡: significant increase

### Intervention characteristics

Details of the interventions are reported in Table 3. Social Cognitive Theory informed intervention development in four studies (5 papers) [29, 33, 43–45], with two of these additionally informed by Family Systems Theory [45] or Self-Determination Theory and Social-Ecological Model [33]. The Theory of Self-Efficacy [47] and Social Learning Theory [31] informed intervention development in one study each. The remaining studies did not specify an underlying theory for intervention development [30, 32, 34, 46]. A range of behavior change techniques were employed across interventions, including goal setting [29, 33, 34, 43, 45–47], behavior substitution [29, 30, 33, 34, 43–47], information about the behavior-health link [29, 33, 34, 43–47], restructuring the home environment [30, 33, 34, 44, 45, 47], behavioral practice [47], and problem solving [29, 32–34, 43–45, 47]. Most interventions focused on reducing duration of screen use [31–34, 44–47], while others focused on context of use including substituting violent content with prosocial/educational content without trying to reduce total screen time (content) [29, 43] or removing screen time in the hour before bed (timing) [30]. Intervention duration ranged from a one-off 10-minute session [46] to twelve months [29, 43]. Approximately half of the interventions (*n* = 6) were delivered weekly [30, 31, 33, 34, 45, 47], with others delivered fortnightly [44] or monthly [29, 32, 43], and one intervention involved a one-off counselling session [46]. Session length ranged from 10-minutes [46] to one-hour [45]. Most interventions (*n* = 8) were delivered in-person [29, 31, 32, 34, 43, 45–47], with others delivered via telephone or video call [30, 44] or via information-based videos and information booklets [33]. Interventions were delivered in the home [29, 30, 34, 43], primary care settings [31, 44, 46], university [45], tertiary care hospital immunization clinic [32], at a place convenient to participants [33] or kindergartens [47].

**Table 3:** Description of intervention characteristics

Study		Behaviour Change Techniques																				
	Theory	AP	BP	BS	CR	Ed	ER	Fe	GS	GR	PR	PS	Re	Rew	SC	SM	Res	Focus and content	Duration and dose	Delivery mode, setting and provider	Assessment timepoints	Comparison group
Birken 2012 (1)	None			x	x	x			x		x			x		x		Focus: screen time reduction Content: Standardised counselling on safe media use and handout from Canadian Paediatric Society titled “Managing Media in the Home”, AND behavioural counselling on the health impact of screen time and strategies to decrease screen time.	One-off, 10-min session	In-person Primary care (During 3-year-old universal health visit) Trained study personnel with graduate-level training in dietetics	1-year (during 4-year-old universal health visit)	Standardised counselling on safe media use and handout from Canadian Paediatric Society titled “Managing Media in the Home”
Bulduk 2025 (2)	SLT					x												Focus: screen time reduction Content: Parent education on the adverse effects of digital device use.	Weekly 20-min sessions for 2 weeks	In-person Primary care (Family Health Centre) Nurses	Post intervention	Wait-list control
Christakis 2013 (3)	SCT	x		x		x		x	x	x		x	x				x	Focus: screen content Content: Parents were assisted to substitute violent content with prosocial/educational content without trying to reduce total screen time.	Monthly (session length not reported) for 12 months	In-person (initial session), followed by mailings and phone calls Home Case manager	6 & 12 months	Nutrition al intervention designed to promote healthy eating
Garrison 2012 (4)																						
Epstein 2008 (5)	None			x		x	x		x					x		x		Focus: screen time reduction Content: TV allowance device (attached to all computers and TV in the home) with weekly time limits set by study staff, plus strategies to reduce sedentary behaviour.	Weekly for 6 months	In-person and newsletters Home Study staff	6, 12, 18, 24 months	Received a newsletter with parenting tips, sample praise statements, and child-appropriate activities and recipes
Hinkley 2015 (6)	SCT FST	x		x		x	x		x	x		x	x			x		Focus: screen time reduction Content: Parent education on strategies to reduce electronic media use, both non-selectively (e.g. by removing screens from bedrooms) and selectively (e.g. by setting rules) and alternatives (e.g. reading, imaginative play)	Weekly 1-hour sessions for 5 weeks	In-person University Trained facilitator, not part of the research team	Post intervention	Wait-list control
Kaur 2024 (7)	SCT SDT SEM			x		x	x	x	x			x		x				Focus: screen time reduction Content: Program to Lower Unwanted Media Screens (PLUMS): Parent education program covering screen-time and child development, household rules, media and sleep, home environment, mealtime media use, family communication, community engagement, and positive reinforcement.	*Parent module* Weekly 30 min sessions for 2 months *Child module* Daily 30–60 min sessions for 2 months	Information based videos and information booklets Place convenient to participants Researcher	Immediately post intervention, 6 months	Usual practice
Lin 2021 (8)	TSE		x	x		x	x	x	x			x	x					Focus: screen time reduction Content: Parental education to increase knowledge and self-efficacy towards screen use and the importance of monitoring and changing their children’s screen behaviours. Intervention involved group discussions, roleplay and reflection.	Weekly 50 min sessions for 8 weeks	In-person Kindergartens Kindergarten administrators	Post intervention	Usual practice
Pickard 2024 (9)	None			x				x										Focus: screen time before bed Content: Parent-Administered Screen Time Intervention (PASTI) Caregivers removed toddler screen time in the hour before bed and used activities from a bedtime box instead, which included tips on alternative before-bed activities (e.g., reading, puzzles).	Weekly for 7 weeks	Phone/video call Home Researcher	Post intervention	Usual practice
Poonia 2024 (10)	None					x												Focus: screen time reduction Content: Active counselling to reduce screen time. Parents were guided to limit screen exposure and modify parental media habits.	30-min initial session + monthly telephone follow-up For 6 months	In-person, printed materials and telephone Tertiary care hospital, immunisation clinic Healthcare professionals	Post intervention	Routine counselling on nutrition, immunisation and safety.
Yilmaz 2015 (11)	SCT			x		x	x	x				x			x			Focus: screen time reduction Content: Parents received printed materials, interactive CD’s and one counselling call on harmful effects of excessive screen time and suggestions for alternative activities, aimed at decreasing screen time.	Fortnightly for 8 weeks	Telephone and printed materials Primary care Healthcare professionals	2, 6, 9 months	Usual practice

*Abbreviations*: *AP *Action planning, *BP *Behaviour practice, *BS *Behaviour substitution, *CR *Cognitive restructuring, *Ed *Education (health impact of screen time), *Fe *Feedback, *GS *Goal setting , *GR *Goal review, *PR *Positive reinforcement, *PS *Problem solving, *Rew *Rewards, *SC *Social comparison, *SM *Self-monitoring, *Res *Resources, *SLT *Social Learning Thory, *SCT *Social Cognitive Theory, *FST *Family Systems Theory, *SDT *Self-determination Theory, *SEM *Social-Ecological Model, *TSE *Theory of Self-Efficacy

### Assessment of methodological quality

Appraisal of study quality against 13 criteria is shown in Table 4 [48]. For all studies treatment groups were treated identically other than the intervention (Table 3, Q7), outcomes were measured in the same way for treatment groups (Table 3, Q10), appropriate statistical analysis was used (Table 3, Q12) and the trial design was appropriate (Table 3, Q13). Across most studies a true randomization procedure for group allocation was employed (82%; Table 3, Q1), participants were analysed in the groups they were allocated (91%; Table 3, Q9) and outcomes were measured in a reliable way (73%; Table 3, Q11).However, few studies concealed group allocation (36%; Table 3, Q2)or specified blinding of participants (18%; Table 3, Q4), or outcome assessors (45%; Table 3, Q6) and none were able to blind intervention providers (0%; Table 3, Q5). Finally, although studies reported the number of participants lost to follow up, few (18%) reported the reasons for attrition or assessed its potential impact on study outcomes (Table 3, Q8).

**Table 4:** Assessment of methodological quality of included studies

Study	Q1	Q2	Q3	Q4	Q5	Q6	Q7	Q8	Q9	Q10	Q11	Q12	Q13	% Yes
Birken 2012	Y	Y	Y	Y	N	Y	Y	N	Y	Y	U	Y	Y	77%
Bulduk 2025	Y	U	N	U	U	U	Y	Y	Y	Y	N	Y	Y	54%
Christakis 2013	U	U	Y	N	N	U	Y	N	Y	Y	Y	Y	Y	54%
Epstein 2008	Y	U	Y	U	N	U	Y	N	Y	Y	Y	Y	Y	62%
Garrison 2012	Y	U	Y	N	N	U	Y	N	Y	Y	Y	Y	Y	62%
Hinkley 2015	Y	Y	Y	U	U	Y	Y	Y	Y	Y	U	Y	Y	77%
Kaur 2024	Y	U	Y	N	N	Y	Y	N	Y	Y	Y	Y	Y	62%
Lin 2021	U	Y	Y	N	N	N	Y	N	Y	Y	Y	Y	Y	62%
Pickard 2024	Y	U	Y	Y	U	Y	Y	N^a^	Y	Y	Y	Y	Y	77%
Poonia 2024	Y	Y	N	N	N	N	Y	N	Y	Y	Y	Y	Y	62%
Yilmaz 2015	Y	U	Y	N	N	Y	Y	N	U	Y	Y	Y	Y	62%
% Yes	82%	36%	73%	18%	0%	45%	100%	18%	91%	100%	73%	100%	100%	

Note: Y, yes; N, no; U, unclear

^a^ one participant lost to follow up (intervention group: *n* = 35; control group: *n* = 34)

Q1: Was true randomization used for assignment of participants to treatment groups?

Q2: Was allocation to treatment groups concealed?

Q3: Were treatment groups similar at the baseline?

Q4: Were participants blind to treatment assignment?

Q5: Were those delivering the treatment blind to treatment assignment?

Q6: Were outcomes assessors blind to treatment assignment?

Q7: Were treatment groups treated identically other than the intervention of interest (e.g., attention-matched control)?

Q8: Was follow-up complete, and if not, were differences between groups in terms of their follow-up adequately described and analysed?

Q9: Were participants analysed in the groups to which they were randomized? (ITT)

Q10: Were outcomes measured in the same way for treatment groups?

Q11: Were outcomes measured in a reliable way?

Q12: Was appropriate statistical analysis used?

Q13: Was the trial design appropriate and any deviations from the standard RCT design (individual randomization, parallel groups) accounted for in

the conduct and analysis of the trial?

%Yes: percentage of studies meeting question criteria

*Studies included in meta-analysis

### Meta-analytic and systematic review results

#### Intervention effects on social-emotional development – all domains combined

**Fig. 2:**
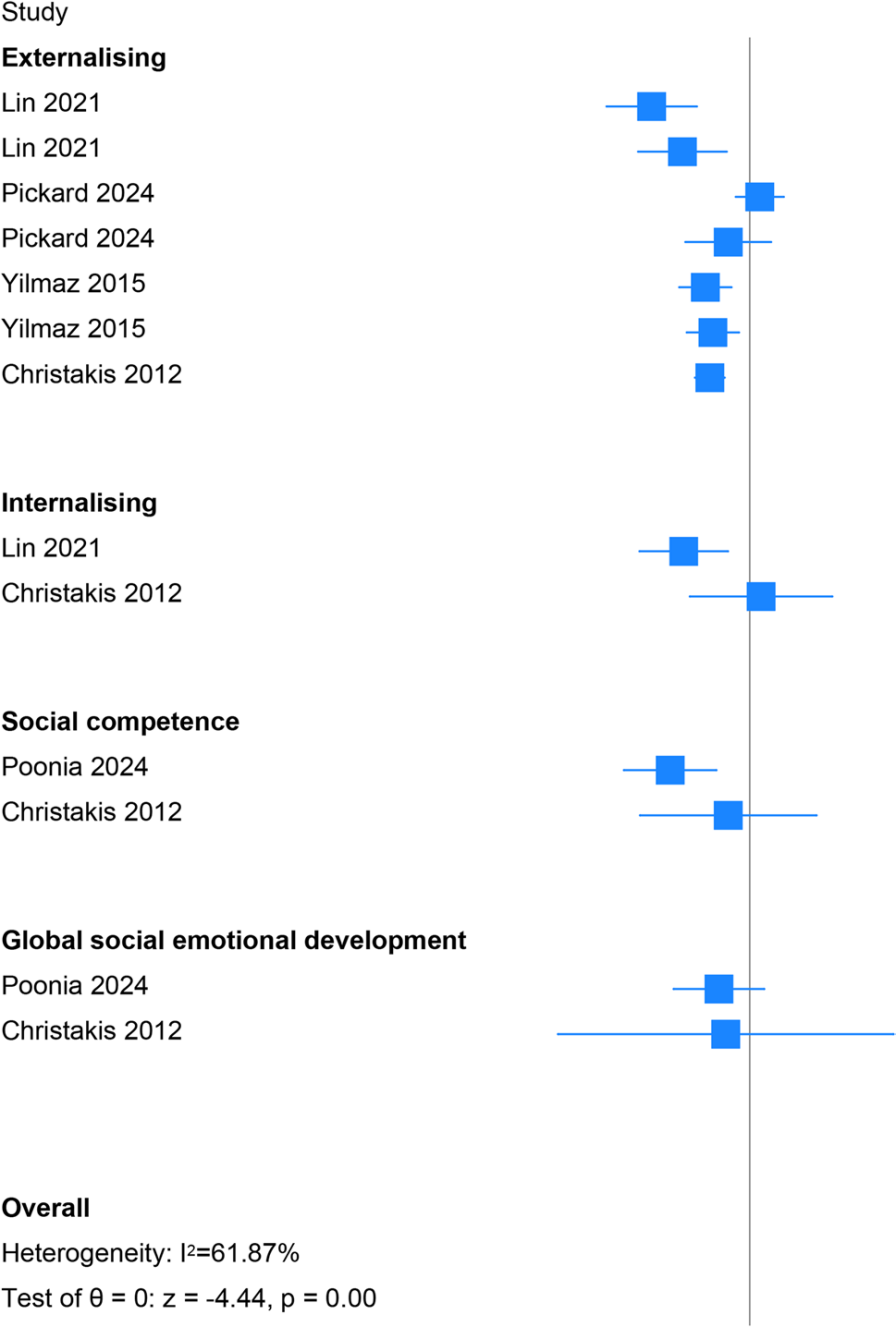
forest plot of intervention effects on social-emotional development

Intervention effects on at least one social-emotional development domain were assessed across six studies [29, 30, 32, 33, 44, 47]. Five studies describing 13 effect sizes (*n* = 1,279) were included in multi-level meta-analysis [29, 30, 32, 44, 47]. The pooled effect size was Cohen’s *d* = -0.32 (95%CI: -0.51 to -0.12), indicating a small reduction in social-emotional problems in favour of the intervention group. Medium heterogeneity was observed (I^2^ = 61.87%) (Fig. 2). The one study that could not be included in the meta-analysis (as it reported the regression coefficient rather than the mean and standard deviation for emotional problem score) indicated the intervention had no effect on emotional problems at either the two or six month follow-up [33]. However, it did lead to a reduction in screen time at two months, although this effect was not maintained at the six month follow-up [33].

### Intervention effects on individual domains of social-emotional development

#### Externalizing behaviors

Intervention effects on externalizing behaviors were assessed across five studies [29, 30, 33, 44, 47], with four included in multi-level meta-analysis, contributing seven effect sizes (*n* = 1159) [29, 30, 44, 47]. As shown in Fig. 2 (above), the pooled effect size was Cohen’s *d* = -0.31 (95%CI: -0.56,-0.05), indicating a small but significant improvement in favor of the intervention group. Substantial heterogeneity was present (*I*^*2*^ = 75.44%). Interventions that were underpinned by a theory and incorporated a range of established behavior change techniques had a larger effect on externalizing behaviors (ES=-0.29,-0.78) [29, 44, 47], compared with interventions that did not report these components (ES = 0.08,-0.17) [30]. Notably, a single intervention targeting content quality showed a similar effect size for improving externalizing behaviors (ES=-0.32) [29] as interventions targeting reductions in screen use duration (ES=-0.29,-0.78) [44, 47]. However, an intervention targeting screen use in the hour before bedtime had no effect on externalizing behaviors (ES = 0.08,-0.17) [30].

### Internalising behaviors

Two studies reported intervention effects on internalizing behaviors [29, 47]. While modifying screen content had no effect on internalizing behaviors [29], reducing total daily screen time led to reductions in internalising problems [47].

### Social competence

Two studies reported intervention effects on social competence, with mixed findings [29, 32]. Increasing screen content quality led to improved social competence [29], while reducing overall duration of screen use had no effect on social competence [32].

#### Global social-emotional development

Two studies reported intervention effects on global social-emotional development. Both indicated interventions led to significant improvement in global social-emotional development [29, 32].

### Intervention effects on cognitive development

Intervention effects on cognitive development were reported in two studies reporting on very different interventions, with mixed findings. While reducing total daily screen time led to improvements in problem solving but not communication skills [32], reducing screen time in the hour before bed had no effect on attention [30].

### Intervention effects on physical development

#### Body mass index

Intervention effects on measured standardized body mass index (zBMI) were reported across three studies [34, 44, 46], however they could not be combined in meta-analyses as the required data could not be accurately extrapolated from the provided figures for one study [34]. Neither a one-off 10-minute session [46] nor an 8-week intervention [44] delivered fortnightly showed an effect on zBMI at 1-year and 9-month follow-ups, respectively. In contrast, a six-month intervention delivered weekly led to significant reductions in zBMI at six- and 12-month, but not 18- or 24-month follow-ups [34].

#### Motor skills

Intervention effects on motor skills were reported in one study. A six-month intervention delivered fortnightly achieved reductions in total daily screen time and led to a significant improvement in fine motor but not gross motor skills [32].

### Intervention effects on physical activity

Intervention effects on physical activity were reported across three studies [33, 34, 45] however, these could not be combined in meta-analysis as one study presented results as a regression coefficient rather than a mean and standard deviation [33]. One intervention achieved an increase in parent reported physical activity [33], while the other two interventions had no effect on device measured physical activity [34, 45].

### Intervention effects on sleep characteristics

Intervention effects on sleep characteristics were reported across five studies but were unable to be included in meta-analyses due to heterogeneity in specific sleep characteristics assessed. Results were mixed: three interventions improved various sleep characteristics, including improved sleep quality [47], increased sleep duration, decreased night awakenings and decreased sleep onset latency [31], and decreased sleep problems [43]. These improvements were accompanied by reductions in total screen time [31, 47] and an increase in content quality [43]. One intervention had no effect on sleep duration, night awakenings, sleep efficiency or sleep onset latency [30] despite achieving reductions in screen time in the hour before bed [30]. One intervention had no effect on either sleep problems or screen time duration [33].

### Intervention effects on screen use

**Fig. 3:**
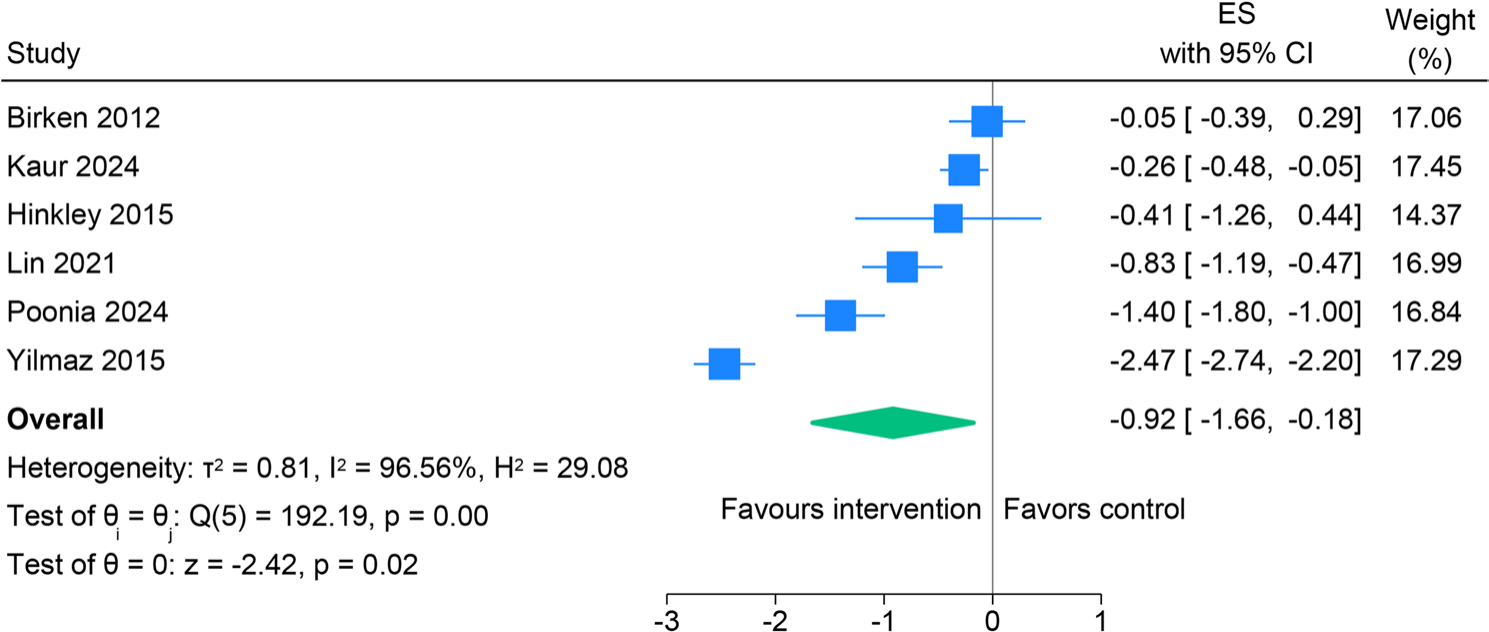
forest plot of intervention effects on screen use

A pooled effect size of Cohen’s *d* = -0.92 (95%CI: -1.66 to -0.18) was observed across six studies (*n* = 1,106) evaluating the impact of interventions on screen time duration [32, 33, 44–47] (Fig. 2), equating to a mean difference of 38 min of screen use per day. However, substantial heterogeneity was present (I^2^ = 96.56%). Larger effect sizes were observed when interventions were underpinned by a theory and incorporated a range of established behavior change techniques, including action planning, behavior substitution, behavior practice (e.g. roleplay), environmental restructuring, goal setting, feedback (ES=-0.26 to -2.47) [33, 44, 45, 47] compared to when interventions did not include these components (ES = 0.05) [46]. One study that did not report on any of these components had a very large effect size (ES=-1.40) [32]. However, given the lack of detailed reporting it is unclear whether any of these features were present. Among four additional studies not included in the meta-analysis, all were effective for modifying screen use behaviors, including duration [31, 34], content (quality) [29, 43], and timing (before bedtime) [30] (Fig. 3).

### Feasibility outcomes

Intervention feasibility was reported in two studies [30, 45]. Hinkley and colleagues’ [45] 5-week pilot RCT to reduce electronic media use was considered feasible and acceptable by parents, with recruitment targets met, 100% participant retention, participants reporting enjoyment of the program and sessions delivered as planned. Similarly, Pickard and colleagues’ [30] 7-week pilot RCT targeting screen use before bedtime demonstrated 99% participant retention and 85% of participants reported the intervention was easy to adhere to.

## Discussion

This is the first systematic review to evaluate the effect of parent-focused interventions for screen use on a broad range of developmental outcomes and screen use behaviors in early childhood. Our analyses revealed four main findings. First, parent-focused interventions for screen use can achieve small reductions in social-emotional problems and externalizing behaviors. Second, such interventions can achieve large reductions in daily screen time duration. Third, interventions underpinned by a theory and incorporating a range of established behavior change techniques were more effective for reducing screen time and improving externalising behaviors compared to interventions that did not include these components. Fourth, there were few studies exploring intervention effects on physical activity, zBMI, cognitive development and/or motor skills, and heterogenous sleep outcomes were reported, making it difficult to make solid conclusions regarding intervention effects on these outcomes. Lastly, there were substantial methodological limitations across included studies.

Our results suggest parent-focused interventions for screen use can achieve small reductions in social emotional problems and externalising behaviors. Notably, a single intervention targeting content quality (replacing violent content with prosocial content) showed a similar effect size for improving externalising behaviors as interventions targeting reductions in screen use duration, warranting further attention. While there is evidence from observational studies showing content quality may be linked to child behaviour [8], further intervention research beyond the single study identified for this review is warranted to confirm the finding. Additionally, future intervention studies may collect feedback from parents on the feasibility and acceptability of targeting content quality (or other screen use contexts) to compare with the feasibility and acceptability of strategies that focus on reducing screen time. Lastly, although observed effects for social-emotional problems and externalising behaviours were small (ES = 0.31–0.32), this equates to an improvement of approximately 12.2–12.6 percentile points (U_3_ = 62.2–62.6) [49], potentially shifting a child from the borderline (> 80th percentile) to the normal range (< 80th percentile), or from the abnormal range (> 90th percentile) to the borderline range [50]. In practical terms, these improvements may reflect children being better able to manage frustration, persist with challenging tasks, and interact positively with peers, parents and caregivers [51]. For families, such improvements may translate to fewer daily behavioural difficulties and lower levels of parental stress. If sustained over time, these improvements in social-emotional development may contribute to improved health, wellbeing, social and educational outcomes [52].

Results from our review show parent-focused interventions for screen use achieved large reductions in screen time in young children (38 min/day). As children aged 2- to 4- years engage in two hours of screen time/day on average [4], this reduction of 38 min/day of screen time represents more than one quarter of average use and potentially brings children into closer alignment with national and international screen time guidelines (≤ 1-hour/day for 2- to 5- year olds) [12, 13]. This time could be reallocated to more cognitively or socially enriching activities such as reading aloud, social interaction, physical activity, play and sleep which may in turn provide beneficial impacts on health and development. is consistent with prior systematic reviews which reported such interventions achieved reductions in screen time of between 13 and 65 min/day [10, 23, 53, 54]. with larger effects for interventions delivered over shorter durations (< 12 weeks; SMD = 0.15) and smaller effects for interventions delivered over longer durations (> 52 weeks; SMD = 0.061) [53].

Interventions appeared most effective for reducing screen use duration and improving externalising behaviors when they incorporated a range of established behavior change techniques and were underpinned by theory, compared with interventions that did not report these components. Specifically, the behavior change techniques of action planning, behavior substitution, behavior practice (e.g. roleplay), environmental restructuring, goal setting, and/or feedback appeared particularly important for successful interventions. These findings are consistent with previous reviews that identified interventions for reducing children’s screen time that included techniques such as goal setting, planning, and feedback had larger effects compared to interventions that did not include these techniques [53, 55]. Behavior practice, behavior substitution, and restructuring of the home environment have also been identified as active ingredients in interventions targeting reductions in children’s screen time [55]. While these components were associated with enhanced intervention effectiveness, there was variability across interventions in how these techniques were delivered during the intervention. However, common among all effective interventions was the inclusion of environmental restructuring and/or goal setting. Therefore, these techniques may provide a starting point from which actionable steps can be taken to promote healthy screen use behavior. While these findings have important implications for the content and design of future interventions, other intervention characteristics, such as intervention length, should also be considered. However, this was not possible in this review due to the limited number of included studies.

With only one to two studies each for cognitive development and motor skill outcomes, and a heterogenous range of sleep outcomes assessed, it is not possible to draw definitive conclusions regarding intervention effects on these outcomes. Intervention effects on physical activity were mixed, with results indicating interventions had no effect on device measured physical activity [34, 45] but led to increases in parent-reported physical activity [33]. Thus, reported intervention effectiveness may be a function of measurement method rather than of the intervention. Studies have shown parents overestimate their child’s physical activity by up to three times, compared to accelerometry [56, 57] and it is possible that parents participating in a physical activity intervention may further inflate their estimates due to social desirability bias. A further possible explanation may relate to the displacement hypothesis, which posits that an decrease in screen time leaves more time available for physical activity [58]. Our results provide support for this notion, indicating an intervention that led to reductions in screen time also led to increased physical activity [33], whereas an intervention that had no effect on screen time also had no effect on physical activity [45]. Consistent with results from prior meta-analyses [56, 57], our results suggest interventions aimed at reducing screen time generally had no effect on zBMI. However, intervention effects may be dependent on intervention duration. A one-off session [46] and an eight-week intervention [44] had no effect on zBMI, while a six-month intervention led to significant reductions in zBMI at six and 12-month follow-ups [34], suggesting interventions may need to be of longer duration to impact zBMI.

There were substantial methodological limitations across included studies which may affect the reliability of our findings. Most studies lacked adequate blinding, had unclear procedures for group allocation concealment, and either did not conduct or did not adequately report attrition analysis. Awareness of group allocation may have influenced parent-reported outcomes, as parents may over- or under- report improvements in child behaviours, thereby introducing reporting bias [59]. This notion is supported by our results showing no intervention effect for studies using objective measures of physical activity [34] while increased physical activity in the intervention group observed in studies using parent-reported physical activity measures [33, 45]. Furthermore, few studies provided reasons for loss to follow-up or explored the potential impact on results. This lack of rigorous reporting means we cannot rule out attrition bias, particularly if dropout patterns differed between groups.

This emerging evidence, while limited in amount and robustness, suggests that parent-focused interventions targeting young children’s screen use have potential to reduce screen use duration and improve social-emotional outcomes. Policies are needed to support parent-focused programs that help parents develop the skills needed to reduce their child’s screen time, alongside systems-level approaches, such as requiring digital media companies to disable autoplay as the default setting [60].

### Strengths and limitations

A key strength of this review is its focus on a range of developmental outcomes in early childhood. We included a wide range of developmental outcomes, including their proximal indicators of sleep and physical activity. In addition, we adhered to rigorous systematic review and meta-analysis approaches, including risk of bias assessment. However, this review had some limitations. The most important limitations of our review relate to the small evidence base we had to work with. For example, there were insufficient studies in some developmental domains, and considerable heterogeneity in intervention components making it difficult to draw solid conclusions regarding intervention effects. Furthermore, we included only studies that reported effects on both screen time and at least one developmental outcome. Therefore, our results for intervention effects on screen use are not comprehensive. However, these results can help to explain the observed intervention effects on developmental outcomes. Other limitations include missing data from four studies despite follow-up attempts made, and the lack of blinding of participants and intervention providers. However, this is typically not possible in behavioral trials. Most studies relied on parent-report measures to assess intervention effects which may over or underestimate outcomes and impact intervention effects. Restricting inclusion to English language publications was a further limitation as it may result in the exclusion of relevant studies published in other languages. Lastly, single studies contributed multiple effect sizes for social-emotional development. However, we adjusted for this using multi-level models.

## Conclusion

This review found parent-focused interventions for screen use may be an effective strategy for modifying young children’s screen use behaviors and may positively impact children’s social-emotional development. Notably, we found a single intervention targeting screen content showed promise for improving externalising behaviors of young children, warranting further attention. Additionally, interventions using a wide range of established behavior change techniques and underpinned by a theory appear to have larger intervention effects. While results are limited due to the low number of included studies, they provide an indication as to the potential of parent-focused interventions to target the screen use behaviors of young children and their development. In addition to the limitation of the small evidence base, findings should be interpreted in light of the methodological limitations across included studies which may inflate effect sizes, particularly for parent-reported outcomes.

## Supplementary Information

Additional file 1: PRISMA checklist. PRISMA checklist

Additional file 2: search strategies for each database. search strategies for each database

## Data Availability

Data sharing is not applicable to this article as no datasets were generated or analysed during the current study.

## Abbreviations

PQ: Parent Questionnaire (tool not explicitly named)
BISQ: Brief Infant Sleep Questionnaire
CSHQ: Children’s Sleep Habits Questionnaire
SCBE: Social Competence and Behavior Evaluation
SDSC: Sleep Disturbance Scale for Children
PSCL: Paediatric Symptom Checklist
ECBQ: Early Childhood Behavior Questionnaire
ASQ3: Ages and Stages Questionnaire
ASQ: SE2:Ages and Stages Questionnaire, Social Emotional
DESQ: Digital Screen Exposure Questionnaire
CBCL: Child Behavior Checklist
BMI: Body Mass Index
EM: Electronic Media
PA: Physical activity
$: Significant decrease
#: Significant increase
