# Supplementary material for: Effect of parent-focused interventions for screen use on developmental outcomes in young children: a systematic review and meta-analysis
Source: Int J Behav Nutr Phys Act. 2026 May 20;23:71. doi: 10.1186/s12966-026-01919-8 (PMC13374355; doi:10.1186/s12966-026-01919-8)
Supplement: Supplementary file 1 — Additional file 1: PRISMA checklist. PRISMA checklist [file 12966_2026_1919_MOESM1_ESM.docx]

**Additional file 1: search strategies for each database**

**Database:** SCOPUS

**Date Searched:** 20 March 2025

|  | **Concept** | **Search terms** | **Results (n)** |
| --- | --- | --- | --- |
| S1 | Screen time | ( TITLE ( "sedentary behav*" ) OR ABS ( "sedentary behav*" ) ) | 13,520 |
| S2 |  | ( TITLE ( "digital media" OR "media exposure" OR "media use" OR "media time" OR "screen use" OR "screen time" OR "screen view*" OR “media screen*” ) OR ABS ( "digital media" OR "media exposure" OR "media use" OR "media time" OR "screen use" OR "screen time" OR "screen view*" OR “media screen*” ) ) | 46,338 |
| S3 |  | ( TITLE ( "passive media" OR "passive technology" ) OR ABS ( "passive media" OR "passive technology" ) ) | 824 |
| S4 |  | ( TITLE ( "interactive media" OR "interactive technology" ) OR ABS ( "interactive media" OR "interactive technology" )) | 5,430 |
| S5 |  | ( TITLE ( "mobile phone*" OR "Smart phone" OR cellphone ) OR ABS ( "mobile phone*" OR "Smart phone" OR cellphone ) ) | 85,310 |
| S6 |  | ( TITLE ( television OR tv OR dvd ) OR ABS ( television OR tv OR dvd ) ) | 177,891 |
| S7 |  | ( TITLE ( ipad OR tablet OR "touch screen*" OR "Electronic device" OR "digital device" ) OR ABS ( ipad OR tablet OR "touch screen*" OR "Electronic device" OR "digital device" ) ) | 240,350 |
| S8 |  | ( TITLE ( computer OR laptop ) OR ABS ( computer OR laptop ) ) | 1,624,509 |
| S9 |  | ( TITLE ( "computer gam*" OR "video gam*" OR "electronic gam*" OR "handheld console" OR "handheld device" ) OR ABS ( "computer gam*" OR "video gam*" OR "electronic gam*" OR "handheld console" OR "handheld device" ) ) | 44,771 |
| S10 |  | ( TITLE ( "digital play" OR "digital engagement" ) OR ABS ( "digital play" OR "digital engagement" ) ) | 987 |
| S11 |  | ( TITLE ( facebook OR instagram OR "social media" OR youtube OR internet ) OR ABS ( facebook OR instagram OR "social media" OR youtube OR internet ) ) | 809,044 |
| S12 |  | S1 OR S2 OR S3 OR S4 OR S5 OR S6 OR S7 OR S8 OR S9 OR S10 OR S11 | 2,868,537 |
| S13 | Parent | ( TITLE ( parent* OR caregiver* OR family OR "family-based" OR "parent-focused" OR "home-based" OR "family-involving" OR "parent-administered" ) OR ABS ( parent* OR caregiver* OR family OR "family-based" OR "parent-focused" OR "home-based" OR "family-involving" OR "parent-administered" ) ) | 3,086,132 |
| S14 | Early childhood | ( TITLE ( infant* OR baby OR babies OR toddler* OR preschool* OR pre-school* OR "early childhood" OR "young child*" OR "early year*" OR kindergarten OR child* ) OR ABS ( infant* OR baby OR babies OR toddler* OR preschool* OR pre-school* OR "early childhood" OR "young child*" OR "early year*" OR kindergarten OR child* ) ) | 3,158,601 |
| S15 | Intervention | ( TITLE ( intervention* OR strateg* OR program* OR rct OR "randomi?ed controlled trial" OR "pilot study" OR " randomi?ed clinical trial” OR " randomi?ed clinical study”) OR ABS ( intervention* OR strateg* OR program* OR rct OR "randomi?ed controlled trial" OR "pilot study" OR " randomi?ed clinical trial” OR " randomi?ed clinical study”) ) | 9,990,895 |
|  |  | S12 AND S13 AND S14 AND S15 | 10,265 |
|  |  | Limit to English language and publication from 2007 | 9,701 |

**Database:** ERIC (via EBSCOhost)

**Date Searched:** 20 March 2025

|  | **Concept** | **Search terms** | **Results (n)** |
| --- | --- | --- | --- |
| S1 | Screen time | TI "sedentary behav*" OR AB "sedentary behav*" | 307 |
| S2 |  | TI ( "digital media" OR "media exposure" OR "media use" OR "media time" OR "screen use" OR "screen time" OR "screen view*" OR “media screen*” ) OR AB ( "digital media" OR "media exposure" OR "media use" OR "media time" OR "screen use" OR "screen time" OR "screen view*" OR “media screen*” ) | 3,187 |
| S3 |  | TI ( "passive media" OR "passive technology" ) OR AB ( "passive media" OR "passive technology" ) | 5 |
| S4 |  | TI ( "interactive media" OR "interactive technology" ) OR AB ( "interactive media" OR "interactive technology" ) | 454 |
| S5 |  | TI ("mobile phone*" OR "Smart phone" OR cellphone ) OR AB ( "mobile phone*" OR "Smart phone" OR cellphone ) | 1,287 |
| S6 |  | TI ( television OR tv OR dvd ) OR AB ( television OR tv OR dvd ) | 21,329 |
| S7 |  | TI ( ipad OR tablet OR "touch screen*" OR "Electronic device" OR "digital device" ) OR AB ( ipad OR tablet OR "touch screen*" OR "Electronic device" OR "digital device" ) | 3,378 |
| S8 |  | TI ( computer OR laptop ) OR AB ( computer OR laptop ) | 80,446 |
| S9 |  | TI ( "computer gam*" OR "video gam*" OR "electronic gam*" OR "handheld console" OR "handheld device" ) OR AB ( "computer gam*" OR "video gam*" OR "electronic gam*" OR "handheld console" OR "handheld device" ) | 2,822 |
| S10 |  | TI ( "digital play" OR "digital engagement" ) OR AB ( "digital play" OR "digital engagement" ) | 117 |
| S11 |  | TI ( facebook OR instagram OR "social media" OR youtube OR internet ) OR AB ( facebook OR instagram OR "social media" OR youtube OR internet ) | 29,414 |
| S12 |  | DE “handheld devices” | 10,401 |
| S13 |  | DE television | 5,618 |
| S14 |  | DE internet | 24,459 |
| S15 |  | S1 OR S2 OR S3 OR S4 OR S5 OR S6 OR S7 OR S8 OR S9 OR S10 OR S11 OR S12 OR S13 OR S14 | 145,676 |
| S16 | Parent | TI ( parent* OR caregiver* OR family OR "family-based" OR "parent-focused" OR "home-based" OR "family-involving" OR "parent-administered" ) OR AB ( parent* OR caregiver* OR family OR "family-based" OR "parent-focused" OR "home-based" OR "family-involving" OR "parent-administered" ) | 255,370 |
| S17 |  | DE “parent child relationship” | 28,001 |
| S18 |  | DE “child rearing” | 10,307 |
| S19 |  | DE parents | 13,269 |
| S20 |  | S16 OR S17 OR S18 OR S19 | 232,659 |
| S21 | Early childhood | TI ( infant* OR baby OR babies OR toddler* OR preschool* OR pre-school* OR "early childhood" OR "young child*" OR "early year*" OR kindergarten OR child* ) OR AB ( infant* OR baby OR babies OR toddler* OR preschool* OR pre-school* OR "early childhood" OR "young child*" OR "early year*" OR kindergarten OR child* ) | 148,700 |
| S22 |  | DE "Preschool Children" | 27,737 |
| S23 |  | DE children | 53,270 |
| S24 |  | DE infants | 14,949 |
| S25 |  | DE “young children” | 27,138 |
| S26 |  | S21 OR S22 OR S23 OR S24 OR S25 | 194,182 |
| S27 | Intervention | TI ( intervention* OR strateg* OR program* OR rct OR "randomi?ed controlled trial" OR "pilot study" OR "randomi?ed clinical trial” OR "randomi?ed clinical study” ) OR AB ( intervention* OR strateg* OR program* OR rct OR "randomi?ed controlled trial" OR "pilot study" OR "randomi?ed clinical trial” OR "randomi?ed clinical study”) | 685,819 |
| S28 |  | DE intervention | 59,645 |
| S29 |  | DE “randomized controlled trials” | 1,918 |
| S30 |  | S27 OR S28 OR S29 | 692,954 |
| S31 |  | S15 AND S20 AND S26 AND S30 | 1,462 |
|  |  | Limit to publication from 2007 AND English language | 651 |

**Database:** APA PsycInfo (via EBSCOhost)

**Date Searched:** 20 March 2025

|  | **Concept** | **Search terms** | **Results (n)** |
| --- | --- | --- | --- |
| S1 | Screen time | TI "sedentary behav*" OR AB "sedentary behav*" | 3,776 |
| S2 |  | TI ( "digital media" OR "media exposure" OR "media use" OR "media time" OR "screen use" OR "screen time" OR "screen view*" OR “media screen*” ) OR AB ( "digital media" OR "media exposure" OR "media use" OR "media time" OR "screen use" OR "screen time" OR "screen view*" OR “media screen*” ) | 11,520 |
| S3 |  | TI ( "passive media" OR "passive technology" ) OR AB ( "passive media" OR "passive technology" ) | 25 |
| S4 |  | TI ( "interactive media" OR "interactive technology" ) OR AB ( "interactive media" OR "interactive technology" ) | 623 |
| S5 |  | TI ("mobile phone*" OR "Smart phone" OR cellphone ) OR AB ( "mobile phone*" OR "Smart phone" OR cellphone ) | 5,521 |
| S6 |  | TI ( television OR tv OR dvd ) OR AB ( television OR tv OR dvd ) | 25,263 |
| S7 |  | TI ( ipad OR tablet OR "touch screen*" OR "Electronic device" OR "digital device" ) OR AB ( ipad OR tablet OR "touch screen*" OR "Electronic device" OR "digital device" ) | 8,586 |
| S8 |  | TI ( computer OR laptop ) OR AB ( computer OR laptop ) | 86,703 |
| S9 |  | TI ( "computer gam*" OR "video gam*" OR "electronic gam*" OR "handheld console" OR "handheld device" ) OR AB ( "computer gam*" OR "video gam*" OR "electronic gam*" OR "handheld console" OR "handheld device" ) | 9,837 |
| S10 |  | TI ( "digital play" OR "digital engagement" ) OR AB ( "digital play" OR "digital engagement" ) | 212 |
| S11 |  | TI ( facebook OR instagram OR "social media" OR youtube OR internet ) OR AB ( facebook OR instagram OR "social media" OR youtube OR internet ) | 78,743 |
| S12 |  | DE “smartphones” | 3,987 |
| S13 |  | DE television | 8,935 |
| S14 |  | DE internet | 40,027 |
| S15 |  | DE “sedentary behavior” | 3,440 |
| S16 |  | DE “screen time” | 1,360 |
| S17 |  | S1 OR S2 OR S3 OR S4 OR S5 OR S6 OR S7 OR S8 OR S9 OR S10 OR S11 OR S12 OR S13 OR S14 OR S15 OR S16 | 225,019 |
| S18 | Parent | TI ( parent* OR caregiver* OR family OR "family-based" OR "parent-focused" OR "home-based" OR "family-involving" OR "parent-administered" ) OR AB ( parent* OR caregiver* OR family OR "family-based" OR "parent-focused" OR "home-based" OR "family-involving" OR "parent-administered" ) | 692,527 |
| S19 |  | DE parenting | 26,797 |
| S20 |  | DE “parent child relations” | 34,716 |
| S21 |  | DE parents | 70,702 |
| S22 |  | DE “parenting interventions” | 700 |
| S23 |  | S18 OR S19 OR S20 OR S21 OR S22 | 697,748 |
| S24 | Early childhood | TI ( infant* OR baby OR babies OR toddler* OR preschool* OR pre-school* OR "early childhood" OR "young child*" OR "early year*" OR kindergarten OR child* ) OR AB ( infant* OR baby OR babies OR toddler* OR preschool* OR pre-school* OR "early childhood" OR "young child*" OR "early year*" OR kindergarten OR child* ) | 890,282 |
| S25 |  | DE "Preschool students" | 14,479 |
| S26 |  | DE “early childhood development” | 18,491 |
| S27 |  | DE “infant development” | 23,147 |
| S28 |  | S24 OR S25 OR S26 OR S27 | 891,919 |
| S29 | Intervention | TI ( intervention* OR strateg* OR program* OR rct OR "randomi?ed controlled trial" OR "pilot study" OR "randomi?ed clinical trial” OR "randomi?ed clinical study”) OR AB ( intervention* OR strateg* OR program* OR rct OR "randomi?ed controlled trial" OR "pilot study" OR "randomi?ed clinical trial” OR "randomi?ed clinical study”) | 1,241,611 |
| S30 |  | DE intervention | 96,154 |
| S31 |  | DE “randomized controlled trials” | 1,149 |
| S32 |  | S29 OR S30 OR S31 | 1,245,563 |
|  |  | S17 AND S23 AND S28 AND S32 | 4,730 |
|  |  | Limit to publication from 2007 AND English language | 3,918 |

**Database:** CINAHL Complete (via EBSCOhost)

**Date Searched:** 20 March 2025

|  | **Concept** | **Search terms** | **Results (n)** |
| --- | --- | --- | --- |
| S1 | Screen time | TI "sedentary behav*" OR AB "sedentary behav*" | 5,787 |
| S2 |  | TI ( "digital media" OR "media exposure" OR "media use" OR "media time" OR "screen use" OR "screen time" OR "screen view*" OR “media screen*” ) OR AB ( "digital media" OR "media exposure" OR "media use" OR "media time" OR "screen use" OR "screen time" OR "screen view*" OR “media screen*” ) | 7,039 |
| S3 |  | TI ( "passive media" OR "passive technology" ) OR AB ( "passive media" OR "passive technology" ) | 8 |
| S4 |  | TI ( "interactive media" OR "interactive technology" ) OR AB ( "interactive media" OR "interactive technology" ) | 218 |
| S5 |  | TI ("mobile phone*" OR "Smart phone" OR cellphone ) OR AB ( "mobile phone*" OR "Smart phone" OR cellphone ) | 5,177 |
| S6 |  | TI ( television OR tv OR dvd ) OR AB ( television OR tv OR dvd ) | 13,409 |
| S7 |  | TI ( ipad OR tablet OR "touch screen*" OR "Electronic device" OR "digital device" ) OR AB ( ipad OR tablet OR "touch screen*" OR "Electronic device" OR "digital device" ) | 14,575 |
| S8 |  | TI ( computer OR laptop ) OR AB ( computer OR laptop ) | 51,853 |
| S9 |  | TI ( "computer gam*" OR "video gam*" OR "electronic gam*" OR "handheld console" OR "handheld device" ) OR AB ( "computer gam*" OR "video gam*" OR "electronic gam*" OR "handheld console" OR "handheld device" ) | 3,766 |
| S10 |  | TI ( "digital play" OR "digital engagement" ) OR AB ( "digital play" OR "digital engagement" ) | 76 |
| S11 |  | TI ( facebook OR instagram OR "social media" OR youtube OR internet ) OR AB ( facebook OR instagram OR "social media" OR youtube OR internet ) | 58,374 |
| S12 |  | MH Smartphone | 5,357 |
| S13 |  | MH Television | 10,539 |
| S14 |  | MH Internet | 54,875 |
| S15 |  | MH sedentary behavior | 11,366 |
| S16 |  | MH screen time | 1,742 |
| S17 |  | MM screen time | 843 |
| S18 |  | MM "Screen Time In Infancy and Childhood" | 143 |
| S19 |  | S1 OR S2 OR S3 OR S4 OR S5 OR S6 OR S7 OR S8 OR S9 OR S10 OR S11 OR S12 OR S13 OR S14 OR S15 OR S16 OR S17 OR S18 | 199,076 |
| S20 | Parent | TI (parent* OR caregiver* OR family OR "family-based" OR "parent-focused" OR "home-based" OR "family-involving" OR "parent-administered" ) OR AB ( parent* OR caregiver* OR family OR "family-based" OR "parent-focused" OR "home-based" OR "family-involving" OR "parent-administered" ) | 481,595 |
| S21 |  | MH parenting | 22,707 |
| S22 |  | MH parent-child relations | 25,924 |
| S23 |  | MH parents | 55,404 |
| S24 |  | MM parents | 27,627 |
| S25 |  | MM “parents education” | 3,747 |
| S26 |  | S20 OR S21 OR S22 OR S23 OR S24 OR S25 | 503,136 |
| S27 | Early childhood | TI ( infant* OR baby OR babies OR toddler* OR preschool* OR pre-school* OR "early childhood" OR "young child*" OR "early year*" OR kindergarten OR child* ) OR AB ( infant* OR baby OR babies OR toddler* OR preschool* OR pre-school* OR "early childhood" OR "young child*" OR "early year*" OR kindergarten OR child* ) | 733,067 |
| S28 |  | MH Child | 545,660 |
| S29 |  | MH Child, Preschool | 236,250 |
| S30 |  | MH infant | 195,300 |
| S31 |  | S27 OR S28 OR S29 OR S30 | 999,774 |
| S32 | Intervention | TI ( intervention* OR strateg* OR program* OR rct OR "randomi?ed controlled trial" OR "pilot study" OR "randomi?ed clinical trial” OR "randomi?ed clinical study”) OR AB ( intervention* OR strateg* OR program* OR rct OR "randomi?ed controlled trial" OR "pilot study" OR "randomi?ed clinical trial” OR "randomi?ed clinical study”)) | 1,315,761 |
| S33 |  | MH "Randomized Controlled Trials" | 150,098 |
| S34 |  | MM "Early Childhood Intervention" | 4,658 |
| S35 |  | S32 OR S33 OR S34 | 1,378,743 |
| S36 |  | S19 AND S26 AND S31 AND S35 | 4,536 |
| S37 |  | Limit to publication from 2007 AND English language | 4,134 |

**Database:** MEDLINE Complete (via EBSCOhost)

**Date Searched:** 20 March 2025

|  | **Concept** | **Search terms** | **Results (n)** |
| --- | --- | --- | --- |
| S1 | Screen time | TI "sedentary behav*" OR AB "sedentary behav*" | 11,420 |
| S2 |  | TI ( "digital media" OR "media exposure" OR "media use" OR "media time" OR "screen use" OR "screen time" OR "screen view*" OR “media screen*” ) OR AB ( "digital media" OR "media exposure" OR "media use" OR "media time" OR "screen use" OR "screen time" OR "screen view*" OR “media screen*” ) | 13,366 |
| S3 |  | TI ( "passive media" OR "passive technology" ) OR AB ( "passive media" OR "passive technology" ) | 57 |
| S4 |  | TI ( "interactive media" OR "interactive technology" ) OR AB ( "interactive media" OR "interactive technology" ) | 357 |
| S5 |  | TI ("mobile phone*" OR "Smart phone" OR cellphone ) OR AB ( "mobile phone*" OR "Smart phone" OR cellphone ) | 15,058 |
| S6 |  | TI ( television OR tv OR dvd ) OR AB ( television OR tv OR dvd ) | 34,789 |
| S7 |  | TI ( ipad OR tablet OR "touch screen*" OR "Electronic device" OR "digital device" ) OR AB ( ipad OR tablet OR "touch screen*" OR "Electronic device" OR "digital device" ) | 73,159 |
| S8 |  | TI ( computer OR laptop ) OR AB ( computer OR laptop ) | 280,813 |
| S9 |  | TI ( "computer gam*" OR "video gam*" OR "electronic gam*" OR "handheld console" OR "handheld device" ) OR AB ( "computer gam*" OR "video gam*" OR "electronic gam*" OR "handheld console" OR "handheld device" ) | 8,058 |
| S10 |  | TI ( "digital play" OR "digital engagement" ) OR AB ( "digital play" OR "digital engagement" ) | 149 |
| S11 |  | TI ( facebook OR instagram OR "social media" OR youtube OR internet ) OR AB ( facebook OR instagram OR "social media" OR youtube OR internet ) | 120,395 |
| S12 |  | MH Smartphone | 11,295 |
| S13 |  | MH Television | 14,325 |
| S14 |  | MH Internet | 85,366 |
| S15 |  | MH “sedentary behavior” | 15,124 |
| S16 |  | MH “screen time” | 1,569 |
| S17 |  | MM “screen time” | 969 |
| S18 |  | MM "Screen Time In Infancy and Childhood" | 0 |
| S19 |  | S1 OR S2 OR S3 OR S4 OR S5 OR S6 OR S7 OR S8 OR S9 OR S10 OR S11 OR S12 OR S13 OR S14 OR S15 OR S16 OR S17 OR S18 | 601,367 |
| S20 | Parent | TI ( parent* OR caregiver* OR family OR "family-based" OR "parent-focused" OR "home-based" OR "family-involving" OR "parent-administered" ) OR AB ( parent* OR caregiver* OR family OR "family-based" OR "parent-focused" OR "home-based" OR "family-involving" OR "parent-administered" ) | 1,724,919 |
| S21 |  | MH parenting | 23,155 |
| S22 |  | MH parent-child relations | 37,778 |
| S23 |  | MH parents | 88,299 |
| S24 |  | MM parents | 43,025 |
| S25 |  | MM “parents education” | 4,964 |
| S26 |  | S20 OR S21 OR S22 OR S23 Or S24 OR S25 | 1,748,565 |
| S27 | Early childhood | TI ( infant* OR baby OR babies OR toddler* OR preschool* OR pre-school* OR "early childhood" OR "young child*" OR "early year*" OR kindergarten OR child* ) OR AB ( infant* OR baby OR babies OR toddler* OR preschool* OR pre-school* OR "early childhood" OR "young child*" OR "early year*" OR kindergarten OR child* ) | 2,159,288 |
| S28 |  | MH Child | 2,010,343 |
| S29 |  | MH Child, Preschool | 1,028,975 |
| S30 |  | MH infant | 901,383 |
| S31 |  | S27 OR S28 OR S29 OR S30 | 3,330,877 |
| S32 | Intervention | TI ( intervention* OR strateg* OR program* OR rct OR "randomi?ed controlled trial" OR "pilot study" OR "randomi?ed clinical trial” OR "randomi?ed clinical study”) OR AB ( intervention* OR strateg* OR program* OR rct OR "randomi?ed controlled trial" OR "pilot study" OR "randomi?ed clinical trial” OR "randomi?ed clinical study”) | 4,188,486 |
| S33 |  | MH Randomized Controlled Trial | 633,227 |
| S34 |  | MM "Early Childhood Intervention" | 0 |
| S35 |  | S32 OR S33 OR S34 | 4,579,732 |
| S36 |  | S17 AND S22 AND S27 AND S30 | 8,548 |
| S37 |  | Limit to publication from 2007 AND English language | 7,539 |

**Database:** Cochrane Library

**Date Searched:** 20 March 2025

|  | **Concept** | **Search terms** | **Results (n)** |
| --- | --- | --- | --- |
| S1 | Screen time | (sedentary NEXT behav*):ti OR (sedentary NEXT behav*):ab | 2,410 |
| S2 |  | (digital NEXT media OR media NEXT exposure OR media NEXT use OR media NEXT time OR screen NEXT use OR screen NEXT time OR screen NEXT view* OR media NEXT screen*):ti OR (digital NEXT media OR media NEXT exposure OR media NEXT use OR media NEXT time OR screen NEXT use OR screen NEXT time OR screen NEXT view* OR media NEXT screen*):ab | 2,884 |
| S3 |  | (passive NEXT media OR passive NEXT technology):ti OR (passive NEXT media OR passive NEXT technology):ab | 4 |
| S4 |  | (interactive NEXT media OR interactive NEXT technology):ti OR (interactive NEXT media OR interactive NEXT technology):ab | 136 |
| S5 |  | (mobile NEXT phone* OR Smart NEXT phone OR cellphone):ti OR (mobile NEXT phone* OR Smart NEXT phone OR cellphone):ab | 5,982 |
| S6 |  | (television OR tv OR dvd ):ti OR ( television OR tv OR dvd ):ab | 4,063 |
| S7 |  | ( ipad OR tablet OR touch NEXT screen* OR Electronic NEXT device OR digital NEXT device):ti OR ( ipad OR tablet OR touch NEXT screen* OR Electronic NEXT device OR digital NEXT device):ab | 39,454 |
| S8 |  | (computer OR laptop):ti OR (computer OR laptop):ab | 32,807 |
| S9 |  | (computer NEXT gam* OR video NEXT gam* OR electronic NEXT gam* OR handheld NEXT console OR handheld NEXT device):ti OR (computer NEXT gam* OR video NEXT gam* OR electronic NEXT gam* OR handheld NEXT console OR handheld NEXT device):ab | 2,545 |
| S10 |  | (digital NEXT play OR digital NEXT engagement):ti OR (digital NEXT play OR digital NEXT engagement):ab | 23 |
| S11 |  | (facebook OR instagram OR social NEXT media OR youtube OR internet):ti OR (facebook OR instagram OR social NEXT media OR youtube OR internet):ab | 16,689 |
| S12 |  | MeSH descriptor: [Smartphone] this term only | 1223 |
| S13 |  | MeSH descriptor: [Television] this term only | 418 |
| S14 |  | MeSH descriptor: [Internet] this term only | 5480 |
| S15 |  | MeSH descriptor: [sedentary behavior] this term only | 1881 |
| S16 |  | MeSH descriptor: [screen time] this term only | 80 |
| S17 |  | #1 OR #2 OR #3 OR #4 OR #5 OR #6 OR #7 OR #8 OR #9 OR #10 OR #11 OR #12 OR #13 OR #14 OR #15 OR #16 | 102,730 |
| S18 | Parent | (parent* OR caregiver* OR family OR family-based OR parent-focused OR home-based OR family-involving OR parent-administered):ti OR (parent* OR caregiver* OR family OR family-based OR parent-focused OR home-based OR family-involving OR parent-administered):ab | 111,116 |
| S19 |  | MeSH descriptor: [parenting] this term only | 2,159 |
| S20 |  | MeSH descriptor: [parent-child relations] this term only | 1,591 |
|  |  | MeSH descriptor: [parents] this term only | 0 |
| S21 |  | #18 OR #19 OR #20 | 111,228 |
| S22 | Early childhood | (infant* OR baby OR babies OR toddler* OR preschool* OR pre-school* OR early NEXT childhood OR young NEXT child* OR early NEXT year* OR kindergarten OR child*):ti OR (infant* OR baby OR babies OR toddler* OR preschool* OR pre-school* OR early NEXT childhood OR young NEXT child* OR early NEXT year* OR kindergarten OR child*):ab | 210,768 |
| S23 |  | MeSH descriptor: [child] this term only | 71,761 |
| S24 |  | MeSH descriptor: [Child, Preschool] this term only | 39,434 |
| S25 |  | MeSH descriptor: [infant] this term only | 30,462 |
| S26 |  | #22 OR #23 OR #24 OR #25 | 235,247 |
| S27 | Intervention | (intervention* OR strateg* OR program* OR rct OR randomi?ed NEXT controlled NEXT trial OR pilot NEXT study OR randomi?ed NEXT clinical NEXT trial OR randomi?ed NEXT clinical NEXT study):ti OR (intervention* OR strateg* OR program* OR rct OR randomi?ed NEXT controlled NEXT trial OR pilot NEXT study OR randomi?ed NEXT clinical NEXT trial OR randomi?ed NEXT clinical NEXT study):ab | 1,441,684 |
| S28 |  | MeSH descriptor: [Randomized Controlled Trial] this term only | 34 |
|  |  | MM "Early Childhood Intervention" |  |
| S29 |  | #27 OR #28 | 1,441,694 |
| S30 |  | #17 AND #21 AND #26 AND #29 | 5,254 |
| S31 |  | with Cochrane Library publication date from Jan 2007 to present, in Trials | 5,003 |

**Database:** Informit Health Collection

**Date Searched:** 18 March 2025

|  | **Concept** | **Search terms** | **Results (n)** |
| --- | --- | --- | --- |
| S1 | Screen time | (Title:(sedentary behav*) OR Title:“digital media” OR Title:“media exposure” OR Title:“media use” OR Title:“media time” OR Title:“screen use” OR Title:“screen time” OR Title:(screen view*) OR Title:(media screen*) OR Title:“passive media” OR Title:“passive technology” OR Title:“interactive media” OR Title:“interactive technology” OR Title:(mobile phone*) OR Title:“Smart phone" OR Title:Cellphone OR Title:Television OR Title:TV OR Title:DVD OR Title:iPad OR Title:Tablet OR Title:(touch screen*) OR Title:"Electronic device" OR Title:“digital device” OR Title:Computer OR Title:Laptop OR Title:(computer gam*) OR Title:(video gam*) OR Title:(electronic gam*) OR Title:"handheld console" OR Title:“handheld device” OR Title:“digital play” OR Title:“digital engagement” OR Title:Facebook OR Title:Instagram OR Title:“social media" OR Title:Youtube OR Title:Internet) OR (Abstract:(sedentary behav*) OR Abstract:“digital media” OR Abstract:“media exposure” OR Abstract:“media use” OR Abstract:“media time” OR Abstract:“screen use” OR Abstract:“screen time” OR Abstract:(screen view*) OR Abstract:(media screen*) OR Abstract:“passive media” OR Abstract:“passive technology” OR Abstract:“interactive media” OR Abstract:“interactive technology” OR Abstract:(mobile phone*) OR Abstract:“Smart phone" OR Abstract:Cellphone OR Abstract:Television OR Abstract:TV OR Abstract:DVD OR Abstract:iPad OR Abstract:Tablet OR Abstract:(touch screen*) OR Abstract:"Electronic device" OR Abstract:“digital device” OR Abstract:Computer OR Abstract:Laptop OR Abstract:(computer gam*) OR Abstract:(video gam*) OR Abstract:(electronic gam*) OR Abstract:"handheld console" OR Abstract:“handheld device” OR Abstract:“digital play” OR Abstract:“digital engagement” OR Abstract:Facebook OR Abstract:Instagram OR Abstract:“social media" OR Abstract:Youtube OR Abstract:Internet) | 3901 |
| S2 | Parent | (Title:parent* OR Title:caregiver* OR Title:family OR Title:"family-based" OR Title:"parent-focused" OR Title:"home-based" OR Title:"family-involving" OR Title:"parent-administered") OR (Abstract:parent* OR Abstract:caregiver* OR Abstract:family OR Abstract:"family-based" OR Abstract:"parent-focused" OR Abstract:"home-based" OR Abstract:"family-involving" OR Abstract:"parent-administered") | 9236 |
| S3 | Early childhood | (Title:infant* OR Title:baby OR Title:babies OR Title:toddler* OR Title:preschool* OR Title:pre-school* OR Title:"early childhood" OR Title:(young child*) OR Title:(early year*) OR Title:kindergarten OR Title:child*) OR (Abstract:infant* OR Abstract:baby OR Abstract:babies OR Abstract:toddler* OR Abstract:preschool* OR Abstract:pre-school* OR Abstract:"early childhood" OR Abstract:(young child*) OR Abstract:(early year*) OR Abstract:kindergarten OR Abstract:child) | 12,482 |
| S4 | Intervention | (Title:intervention* OR Title:strateg* OR Title:program* OR Title:rct OR Title:"randomi?ed controlled trial" OR Title:"pilot study" OR Title:"randomi?ed clinical trial” OR Title:"randomi?ed clinical study”) OR (Abstract:intervention* OR Abstract:strateg* OR Abstract:program* OR Abstract:rct OR Abstract:"randomi?ed controlled trial" OR Abstract:"pilot study" OR Abstract:"randomi?ed clinical trial” OR Abstract:"randomi?ed clinical study”) | 20,661 |
| S5 |  | S1 AND S2 AND S3 AND S4 | 61 |
|  |  | Limit to English language and publication from 2007 | 54 |

**Database:** Google Scholar

**Date Searched:** 28 March 2025

*****search from 2007**

(facebook OR instagram OR "social media" OR youtube OR internet OR "digital play" OR "digital engagement" OR "computer gam*" OR "video gam*" OR "electronic gam*" OR "handheld console" OR "handheld device" OR computer OR laptop OR ipad OR tablet OR "touch screen*" OR "Electronic device" OR "digital device" OR television OR tv OR dvd OR "mobile phone*" OR "Smart phone" OR cellphone OR "interactive media" OR "interactive technology" OR "passive media" OR "passive technology" OR "digital media" OR "media exposure" OR "media use" OR "media time" OR "screen use" OR "screen time" OR "screen view*" OR "media screen*" OR "sedentary behav*")

AND

(parent* OR caregiver* OR family OR "family-based" OR "parent-focused" OR "home-based" OR "family-involving" OR "parent-administered")

AND

(infant* OR baby OR babies OR toddler* OR preschool* OR pre-school* OR "early childhood" OR "young child*" OR "early year*" OR kindergarten OR child*)

AND

(intervention* OR strateg* OR program* OR rct OR "randomi?ed controlled trial" OR "pilot study" OR "randomi?ed clinical trial" OR "randomi?ed clinical study")
